# Naturally produced type I IFNs enhance human myeloid dendritic cell maturation and IL-12p70 production and mediate elevated effector functions in innate and adaptive immune cells

**DOI:** 10.1007/s00262-018-2204-2

**Published:** 2018-07-13

**Authors:** Annette E. Sköld, Till S. M. Mathan, Jasper J. P. van Beek, Georgina Flórez-Grau, Michelle D. van den Beukel, Simone P. Sittig, Florian Wimmers, Ghaith Bakdash, Gerty Schreibelt, I. Jolanda M. de Vries

**Affiliations:** 1 Department of Tumor Immunology, Radboud Institute for Molecular Life Sciences, Radboudumc, Geert Grooteplein 26/28, 6525 GA Nijmegen, The Netherlands; 2 Department of Oncology-Pathology, Cancer Center Karolinska, Karolinska Institutet, Stockholm, Sweden; 3 Department of Medical Oncology, Radboud Institute for Molecular Life Sciences, Radboudumc, Nijmegen, The Netherlands; 4 Present Address: Allergic Inflammation Discovery Performance Unit, Respiratory Therapy Area, GlaxoSmithKline, Stevenage, United Kingdom

**Keywords:** Dendritic cells, Cytokines, Cell activation, Tumor immunity, Vaccination

## Abstract

There has recently been a paradigm shift in the field of dendritic cell (DC)-based immunotherapy, where several clinical studies have confirmed the feasibility and advantageousness of using directly isolated human blood-derived DCs over in vitro differentiated subsets. There are two major DC subsets found in blood; plasmacytoid DCs (pDCs) and myeloid DCs (mDCs), and both have been tested clinically. CD1c^+^ mDCs are highly efficient antigen-presenting cells that have the ability to secrete IL-12p70, while pDCs are professional IFN-α-secreting cells that are shown to induce innate immune responses in melanoma patients. Hence, combining mDCs and pDCs poses as an attractive, multi-functional vaccine approach. However, type I IFNs have been reported to inhibit IL-12p70 production and mDC-induced T-cell activation. In this study, we investigate the effect of IFN-α on mDC maturation and function. We demonstrate that both recombinant IFN-α and activated pDCs strongly enhance mDC maturation and increase IL-12p70 production. Co-cultured mDCs and pDCs additionally have beneficial effect on NK and NKT-cell activation and also enhances IFN-γ production by allogeneic T cells. In contrast, the presence of type I IFNs reduces the proliferative T-cell response. The mere presence of a small fraction of activated pDCs is sufficient for these effects and the required ratio between the subsets is non-stringent. Taken together, these results support the usage of mDCs and pDCs combined into one immunotherapeutic vaccine with broad immunostimulatory features.

**Electronic supplementary material:**

The online version of this article (10.1007/s00262-018-2204-2) contains supplementary material, which is available to authorized users.

## Introduction

Dendritic cells (DCs) are professional antigen-presenting cells, best known for their ability to activate and polarize naïve T cells [1]. These characteristics have made DCs an interesting target in vaccine development, and several different protocols have been tested clinically [2]. A classical source of DCs used in clinical studies are monocyte-derived DCs (moDCs) [3]. However, recent progress within the field has made it feasible to use naturally circulating blood DCs in therapeutic vaccination against cancer. Both pDCs and CD1c^+^ mDCs have been tested clinically in cancer patients and antigen-specific T-cell responses and prolonged overall survival have been achieved [4, 5]. Using natural blood DCs in cell-based immunotherapy is more beneficial compared to moDCs in many aspects, such as reduced ex vivo culture time, less exhaustion, and high migratory capacity [6].

In general, mDCs are displaying “classical” DC features, such as efficient antigen uptake and presentation, and have the ability to initiate T helper (Th) 1 responses by production of IL-12p70 [7]. Stimulated pDCs release high quantities of IFN-α, a group of type I IFNs important in responses towards viral infections [8] and an important feedback loop to enhance cellular responses towards the detection of nucleic acids [9]. Type I IFNs also have a stimulating effect on innate immune cells, such as NK cells and NKT cells, and these responses are in turn important to identify and eliminate mutated autologous tumor cells [10]. Hence, a vaccine based on the combination of mDCs and pDCs would have the ability to initiate both adaptive and innate anti-tumor immune responses. At the time of their discovery, DCs were referred to as “nature’s adjuvant” [11], and the two subsets might even have additive effects on each other when matured together. Indeed, the responsiveness to TLR ligands has been shown to increase for both subsets when cultured together [12], and IFN-α has been shown to upregulate additional TLRs on both mDCs and pDCs [13]. During viral infection, pDCs are important stimulators of mDCs, which in turn enhances anti-viral responses [14]. Also, in a murine tumor vaccination model, antigen-specific T-cell responses were increased when mDCs and pDCs were activated together rather than separately [15].

On the other hand, there is a risk that mDC–pDC crosstalk would have a negative impact as a combined vaccine product. Type I IFNs have been reported to have dampening effects on both immune and non-immune cells [8, 16]. Although IFN-α is needed in low levels to induce IL-12 production [17], some studies have indicated a negative effect on human mDCs and moDCs. The release of IL-12 has been reported to be inhibited by high doses of type I IFNs [18–20]. However, several studies have shown an increase of IL-12 production from human [21–23] and murine [13, 17, 24] DCs in the presence of IFN-α.

In present study, we investigate the responses of human blood-derived DC subsets either treated with recombinant IFN-α or IL-12p70 or co-cultured at various ratios. The production of cell type-specific cytokines and the expression of maturation markers are evaluated. Additionally, we investigate how these cultures affect both innate and adaptive immune cells. As stimulus, we use protamine–RNA complexes (pR), a previously described TLR7/8 agonist that induces high levels of both IL-12p70 and IFN-α in mDCs and pDCs, respectively [25–28]. This will prevent possible artifact effects previously detected upon combination of different TLR ligands [29] and better resemble the clinical studies on mDCs and pDCs that are being performed with this stimulus (NCT02574377, NCT02993315, and NCT02692976). We demonstrate that IFN-α matures mDCs and increases IL-12p70 production. In addition, we demonstrate that pR-induced mDC maturation is dependent on type I IFN signaling and that co-cultured DCs increase both innate immune cell and T-cell activations, but dampen the proliferative response of T cells. Our study explores the potential of crosstalk between different subsets of human blood-derived DC subsets and clarifies why contradictory effects of type I IFNs previously have been reported in literature. This study supports the combined usage of CD1c^+^ mDCs and pDCs as a cellular anti-cancer immunotherapy, combining the beneficial effects of both subsets into one potent treatment modality.

## Materials and methods

### Reagents

DCs were activated with TLR7/8 ligand imidazoquinoline (R848, 4 µg/ml; Axxora, San Diego, CA) or protamine–RNA complexes (pR, 15 μg/ml) formed in water as previously described [25] by mixing protamine (Protaminehydrochloride MPH 5000 IE/ml, 0.5 μg/ml; Meda Pharma BV, Amstelveen, the Netherlands) and gp100 mRNA (0.5 μg/ml) in a 2:1 ratio. Recombinant IFN-α2a (Roferon^®^-A, 1; 10; and 100 ng/ml, corresponding to ca 1.35 × 10^2^–1.35 × 10^4^ UI/ml; Roche, Basel, Switzerland) and IL-12p70 (0.2–2 ng/ml; BD Biosciences, San Jose, CA) were used to stimulate mDCs and pDCs, respectively, and recombinant IL-3 (10 ng/ml; Cellgenix, Freiburg, Germany) was used as a survival factor for unstimulated pDCs.

To capture intracellular cytokines for flow cytometry, Brefeldin A (10 μg/ml; Sigma-Aldrich, St Louis, MO) was added to the cultured cells 4 or 12 h before analysis.

Isolated PBLs or T cells were activated with Dynabeads human T-activator CD3/CD28 (anti-CD3/anti-CD28 beads, 0.25 × 10^6^ beads/ml; Life Technologies, Carlsbad, CA).

### Cell isolation and culture

PBMCs were isolated from buffy coats (Sanquin, Nijmegen, The Netherlands) by ficoll density centrifugation (Lymphoprep; Axis-Shield PoC AS, Oslo, Norway). Microbead isolation kits (BDCA1^+^ DC and BDCA4^+^ DC isolation kits; Miltenyi Biotec, Bergisch-Gladbach, Germany) were used to isolate CD1c^+^ mDCs and pDCs from PBMCs. For mDCs, CD14^+^ cells were depleted using CD14 microbeads (Miltenyi Biotec) and prior pDCs isolation, PBLs were prepared from PBMCs by plastic adherence for 1 h in RPMI (Life Technologies) supplemented with 2% human serum (Sanquin, Nijmegen, The Netherlands) and 0.5% antibiotic antimycotic (PAA laboratories, Pasching, Austria). Pan-T cells were negatively selected from PBLs using microbead isolation (Miltenyi Biotec).

Purified cells were cultured at 0.5 × 10^6^ cells/ml in X-VIVO-15 medium (Lonza, Basel, Switzerland) supplemented with 2% human serum. For the co-cultures, autologous mDCs and pDCs were mixed in ratios of 1:1 and 5:1, with the total cell concentration kept constant between the co-culture conditions and single-cultured cells.

### Type I IFN blocking experiment

Cells were cultured with a cocktail of three blocking antibodies: anti-IFN-α (5000 NU/ml), anti-IFN-β (5000 NU/ml), and anti-IFNAR1 (20 mg/ml) (all from PBL Biomedical Laboratories, Piscataway, NJ, USA). Samples were pre-treated with the IFN blocking cocktail for 1 h at 37 °C before addition of stimuli.

### ELISA

Cytokines were measured in cell culture supernatants taken at indicated time points and the levels of IL-12p70, IFN-γ (both from Thermo Scientific, Waltham, MA), IFN-α (Bender Medsystems, Vienna, Austria), and TNF-α (BD Biosciences) were assessed with sandwich ELISA.

### Flow cytometry

Freshly isolated mDC and pDC were stained with the following primary mAbs to evaluate phenotype: anti-CD14-FITC (Dako, Glostrup, Denmark), anti-BDCA1-PE (Miltenyi Biotec), anti-BDCA-2-Pe-Cy7 (Biolegend, San Diego, CA) and anti-CD20-APC (eBioscience). The purity of freshly isolated T cells was determined with mAbs anti-CD20-FITC, anti-CD3-PE, and anti-CD56-APC (all BD Biosciences). DC maturation was evaluated with mAbs anti-CD80-PE, anti-CD86-APC, (both BD Biosciences), and anti-CD40-PE (Immunotech, Marseille, France). In DC co-cultures, mDCs and pDCs were identified by mAbs anti-CD11c-FITC (BD Biosciences) and anti-BDCA2-PE-Cy7 (Biolegend). The cell subsets in PBLs were identified with mAbs anti-CD56-APC and anti-CD3-PE-Cy7 (eBiosciences) and their activation was determined with mAbs anti-CD69-FITC and anti-IFN-γ-BV421 (both from BD Biosciences). For the IFN-γ stainings, fluorescence minus one controls were made on positive controls using either a concentration-matched anti-mouse IgG1-BV421 isotype control (eBioscience) or no mAb at all.

For cultured cells, viability was assessed with Fixable Viability Dye eFluor 780 (eBioscience). In the proliferation assays, PBLs or T cells were labeled with carboxyfluorescein diacetate succinimidyl ester (CFSE, 5 μM; Life Technologies). To perform intracellular cytokine staining (ICS), the cells were fixed and permeabilized using a cytofix/cytoperm kit (BD Biosciences).

The cells were acquired on a FACSVerse (BD Biosciences) and the data were analyzed with FlowJo Software (TreeStar Inc., Ashland, OR). Duplicate events were excluded by FCS-A–FCS-H gating. Lymphocytes or DCs were identified by FCS–SSC and subsequently by phenotypic markers.

### Mixed lymphocyte reaction

T-cell proliferation or innate cell activation was determined in mixed lymphocyte reactions (MLRs). DCs were activated overnight in round-bottomed 96-well plates. In some assays, fresh medium was added to the cultures and the cells were pelleted by centrifugation. The medium was discarded and the procedure was repeated two more times. Allogeneic PBLs or T cells were added in a 10:1 or 5:1 ratio, respectively. PBL activation was evaluated after overnight co-culture and T-cell proliferation after 5 days of co-culture.

### Statistical analyses

Statistical relevance was calculated in GraphPad Prism (v5.0; San Diego, CA). To perform multiple variance analyses, ANOVA with Bonferroni’s multiple comparison test was applied. Wilcoxon matched-pairs signed rank tests were performed for paired analyses.

## Results

### Recombinant IFN-α increases the production of IL-12p70 by mDCs and increases pR-induced maturation

**Fig. 1:**
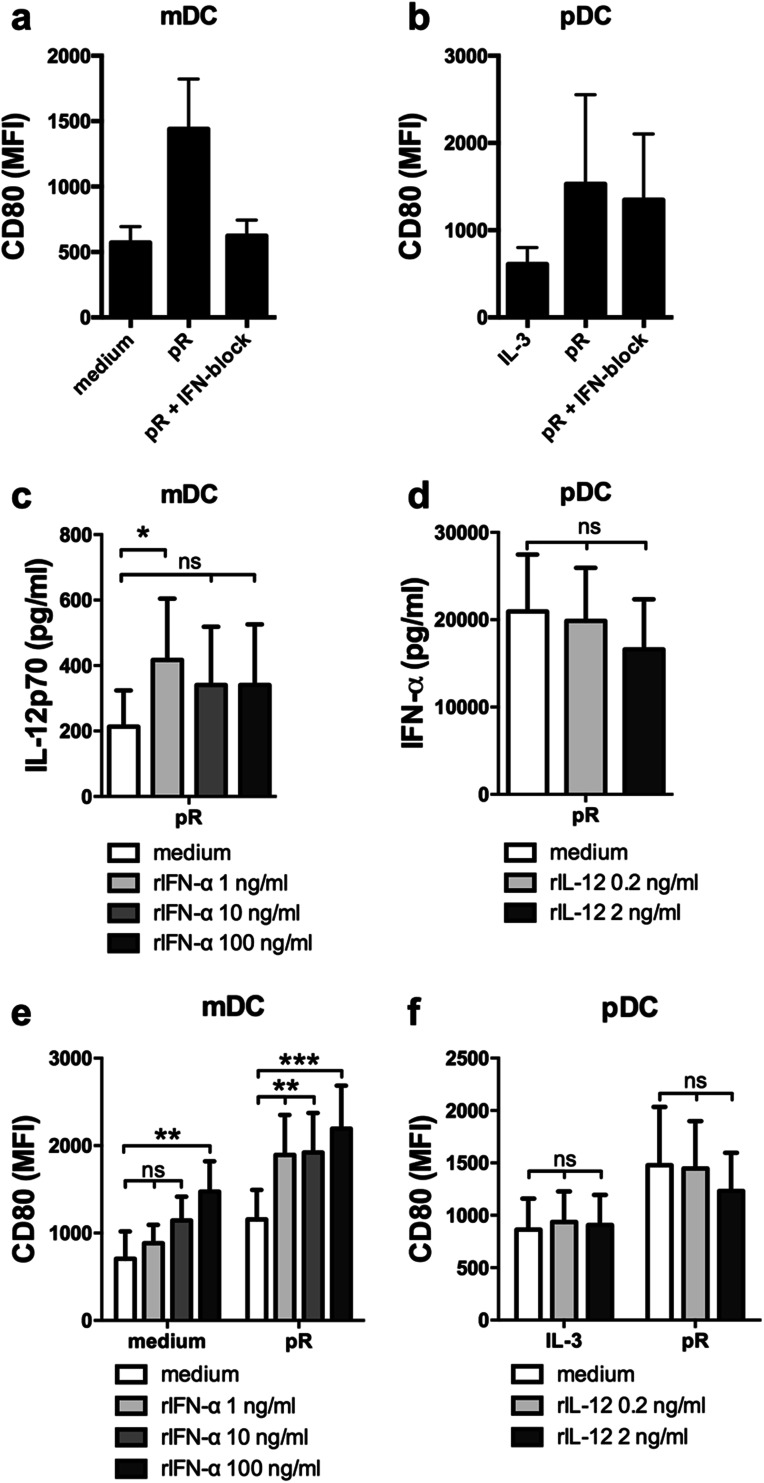
mDCs and pDCs upregulate CD80 and secrete high levels of subset-specific cytokines IL-12p70 and IFN-α, respectively, upon pR treatment. Purified mDCs and pDCs were stimulated overnight with medium alone/IL-3, or pR complexes in the presence or absence of an IFN blocking cocktail or recombinant cytokines. Average expression levels of the CD80 maturation marker ± SEM were measured on **a** mDCs and **b** pDCs from five donors. Cytokine release into supernatant was analyzed with ELISA. Average secreted levels of **c** IL-12p70 ± SEM from seven pR-activated mDC donors in the presence or absence of increasing levels (1; 10; and 100 ng/ml) of rIFN-α and **d** IFN-α ± SEM from nine pR-activated pDC donors in the presence or absence of increasing levels (0.2 and 2 ng/ml) of rIL-12p70. **e** Average MFI expression levels of CD80 ± SEM from seven mDC donors in the presence or absence of increasing levels (1; 10; and 100 ng/ml) of rIFN-α. **f** Average MFI expression levels of CD80 ± SEM from six pDC donors in the presence or absence of increasing levels (0.2 and 2 ng/ml) of rIL-12p70. Statistical differences compared to untreated controls were analyzed by paired one-way ANOVA with Bonferroni’s multiple comparison test and significance is indicated by *(*p* < 0.05), **(*p* < 0.01), ***(*p* < 0.001), or ns (non-significant)

Detection of nucleic acids by endosomal TLRs induces low levels of type I IFN, which in an autocrine manner enhances the response to the ligand [13, 30], and RNA-based adjuvants have clinically been shown to induce type I IFN-mediated anti-tumor responses [9, 31]. We have previously shown that the TLR7/8 ligand protamine–RNA (pR) induces maturation and release of high levels of IL-12p70 and IFN-α by mDCs and pDCs, respectively [25]. We, therefore, first investigated the involvement of type I IFN signaling in pR-mediated DC maturation. DCs were pre-treated with a cocktail of type I IFN blocking antibodies before addition of pR complexes and upregulation of the maturation marker CD80 was investigated after overnight culture. Type I IFN signaling was required for pR-mediated mDC maturation, but not for pDCs (Fig. 1a, b).

Next, we investigated how the DC-specific cytokine production was affected upon addition of recombinant cytokines. To represent both low- and high-producing donors as well as varying DC concentrations, a range between 0.2 and 2 ng/ml of recombinant IL-12p70 (rIL-12) and 1-100 ng/ml rIFN-α (corresponding to ca 1.35 × 10^2^–1.35 × 10^4^ UI/ml) was used in subsequent experiments. An increase of IL-12p70 was detected from pR-stimulated mDCs in the presence of rIFN-α, compared to untreated pR-stimulated cells (Fig. 1c). This is in line with previous observations in moDCs [17]. Higher concentrations of IFN-α had no additive effect, indicating that only minute amounts of IFN-a are necessary to drive this effect. For rIL-12-treated pDCs, no changes in IFN-α production were detected, compared to untreated cells (Fig. 1d).

The expression of maturation marker CD80 and production of the pro-inflammatory cytokine TNF-α was thereafter investigated on cytokine-treated DCs. The presence of rIFN-α had a dose-dependent effect on both pR-stimulated and untreated mDCs (Fig. 1e). Already the lowest dose of rIFN-α significantly elevated the expression of CD80 on pR-stimulated cells, while the highest concentrations were required to mature unstimulated mDCs. TNF-α levels were on the contrary not elevated by rIFN-α (Suppl. Figure 1a). For pDCs, rIL-12 had no maturing effect, neither on IL-3-treated nor pR-stimulated cells (Fig. 1f), and no significant difference in TNF-α production was detected (Suppl. Figure 1b).

### Co-culture of mDCs and pDCs increases the maturation of mDCs, but not pDCs

**Fig. 2:**
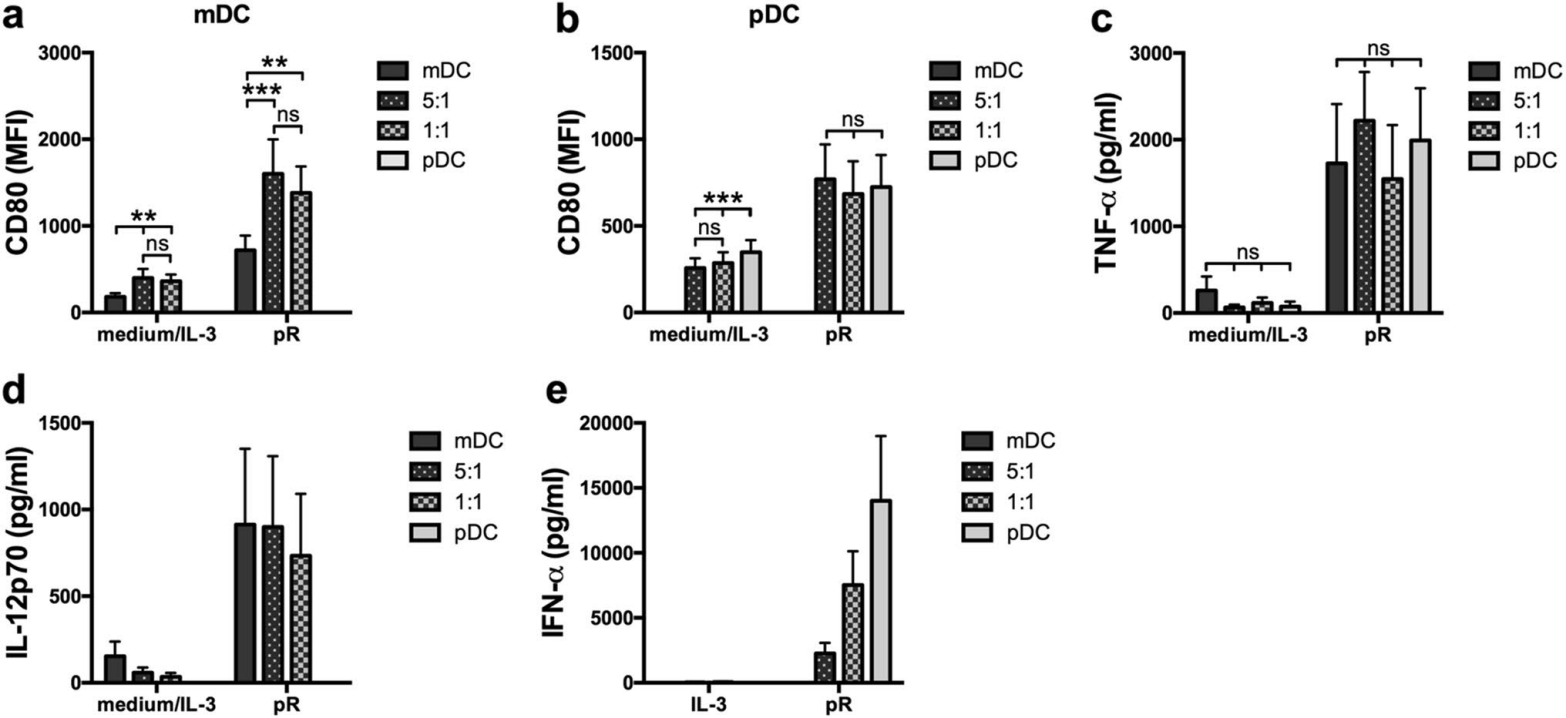
Stimulation of co-cultured autologous mDCs and pDCs enhance pR-mediated mDC, but not pDC, maturation. Purified autologous mDCs and pDCs were cultured overnight either alone or together in a 5:1 or 1:1 ratio in the presence of medium alone/IL-3 or pR complexes. The total number of cells was kept constant between the cultures. Upregulation of maturation marker CD80 was evaluated by flow cytometry. Cytokine release was analyzed with ELISA. **a** mDCs or **b** pDCs from 11 donors cultured either alone or combined at a 5:1 or 1:1 ratio in the presence or absence of stimuli. Average secreted levels of **c** TNF-α ± SEM from eight donors, **d** IL-12p70 ± SEM from seven donors, and **e** IFN-α ± SEM from ten donors. Statistical differences within the treatment groups were analyzed by paired one-way ANOVA with Bonferroni’s multiple comparison test and significance is indicated by **(*p* < 0.01), ***(*p* < 0.001), or ns (non-significant)

Next, the direct impact on co-cultured DC subsets was investigated. Autologous mDCs and pDCs were cultured either alone or in combination in a 5:1 or 1:1 ratio. The total number of cells was kept constant between the different conditions and the cells were either left untreated or stimulated with pR complexes overnight. An increase in expression of maturation markers CD80 and CD86 was detected for the co-cultured pR-stimulated mDCs, compared to mDCs cultured alone (Fig. 2a and Suppl. Figure 2). An increase was also observed in the untreated control, which could be due to the presence of IL-3 in the pDC culture medium. No effect was detected for the pR-treated pDCs (Fig. 2b). There were also no significant changes in TNF-α levels between the different conditions (Fig. 2c). When investigating the secretion of subset-specific cytokines, the ratios of IFN-α corresponded well with the ratio of pDCs, while the IL-12p70 levels were high also in the co-cultured conditions where the ratio of mDCs was less (Fig. 2d, e). This supports the positive effect of IFN-α on IL-12p70 production noted in Fig. 1c.

### The presence of rIFN-α reduces mDC-induced T-cell proliferation, but increases IFN-γ production in innate immune cells

**Fig. 3:**
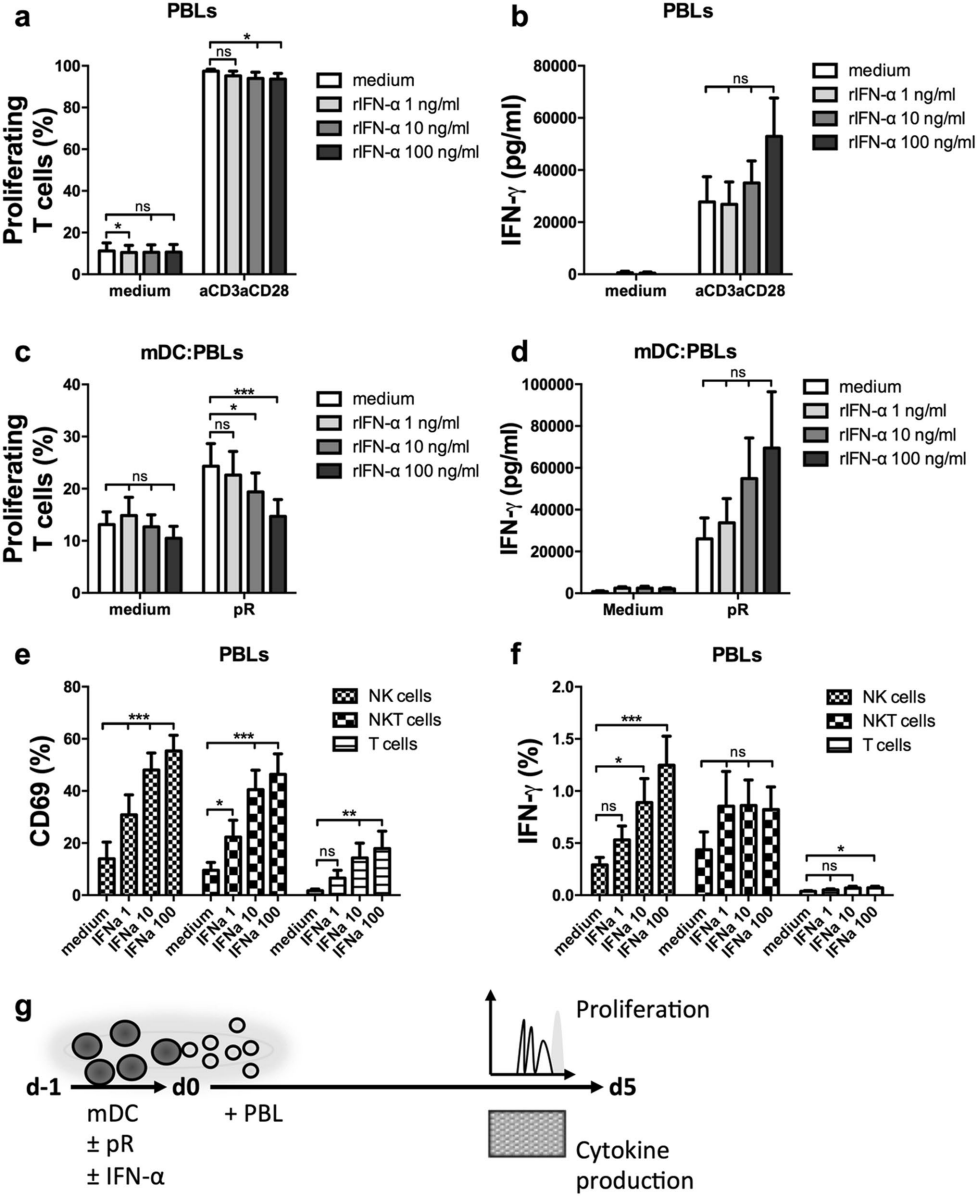
IFN-α reduces T-cell proliferation but activates innate immune cells and increases the production of IFN-γ. PBLs were activated by anti-CD3/anti-CD28 beads or matured allogeneic mDCs in the presence or absence of increasing levels (1; 10; and 100 ng/ml) rIFN-α and the proliferative responses of T cells, or activation of NK, NKT, and T cells were measured by flow cytometry. The release of IFN-γ was measured with ELISA. **a** Mean percentage ± SEM proliferating T cells from nine PBL donors at day 5 of culture. **b** Mean levels of secreted IFN-γ ± SEM from nine PBL donors at day 4 of culture. **c** Mean percentage ± SEM of proliferating T cells at day 5 induced by nine mDC donors. **d** Mean levels of secreted IFN-γ ± SEM at day 4 of co-culture induced by five mDC donors. Mean percentage ± SEM NK, NKT, or T cells expressing **e** the activation marker CD69 or **f** intracellular IFN-γ after overnight treatment in the presence or absence of increasing levels (1; 10; and 100 ng/ml) rIFN-α from nine PBL donors. **g** Illustrative drawing of experimental set-up. Statistical differences compared to untreated controls within each treatment group or for each investigated cell type were analyzed by paired one-way ANOVA with Bonferroni’s multiple comparison test and significance is indicated by *(*p* < 0.05), **(*p* < 0.01), ***(*p* < 0.001), or ns (non-significant)

To evaluate the functional effect of the increased mDC maturation in the presence of rIFN-α, the proliferative T-cell response was investigated in a MLR setting. First, the effect of rIFN-α on PBLs alone was evaluated (Fig. 3a). Anti-CD3/anti-CD28 stimulated T cells displayed a small decrease in proliferation for the higher concentrations. rIFN-α did not significantly affect IFN-γ secretion, but a dose-dependent increase could be detected for most donors (Fig. 3b). When the PBLs were instead stimulated with mDCs activated overnight in the presence or absence of increasing concentration rIFN-α, the dose-dependent decrease in T-cell proliferation was again observed (Fig. 3c, g). A prominent increase of IFN-γ secretion was observed in rIFN-α cultures for most pR-stimulated donors, indicating that rIFN-α promotes cytokine secretion over T-cell proliferation (Fig. 3d, g).

IFN-γ is secreted by activated lymphocytes and acts in a feedback loop to further stimulate Th1 polarization and effector functions [32]. Since IFN-α is known to activate innate immune cells [10, 33], the activation status of natural Killer (NK) cells and NKT cells in the presence of rIFN-α was investigated. PBLs were cultured overnight in the presence of increasing concentrations of rIFN-α, and the upregulation of activation marker CD69 and cell type-specific production of IFN-γ was evaluated for T cells, NK cells, and NKT cells (Fig. 3e, f and Suppl. Figure 3). rIFN-α induced dose-dependent upregulation of CD69 in all cell types investigated (Fig. 3e). Furthermore, it induced IFN-γ production by NK cells (Fig. 3f). Hence, the presence of IFN-α-activated innate immune cells in the MLR of Fig. 3c might affect the observed T-cell responses.

### Innate immune cells are activated in the presence of pR-stimulated pDCs

**Fig. 4:**
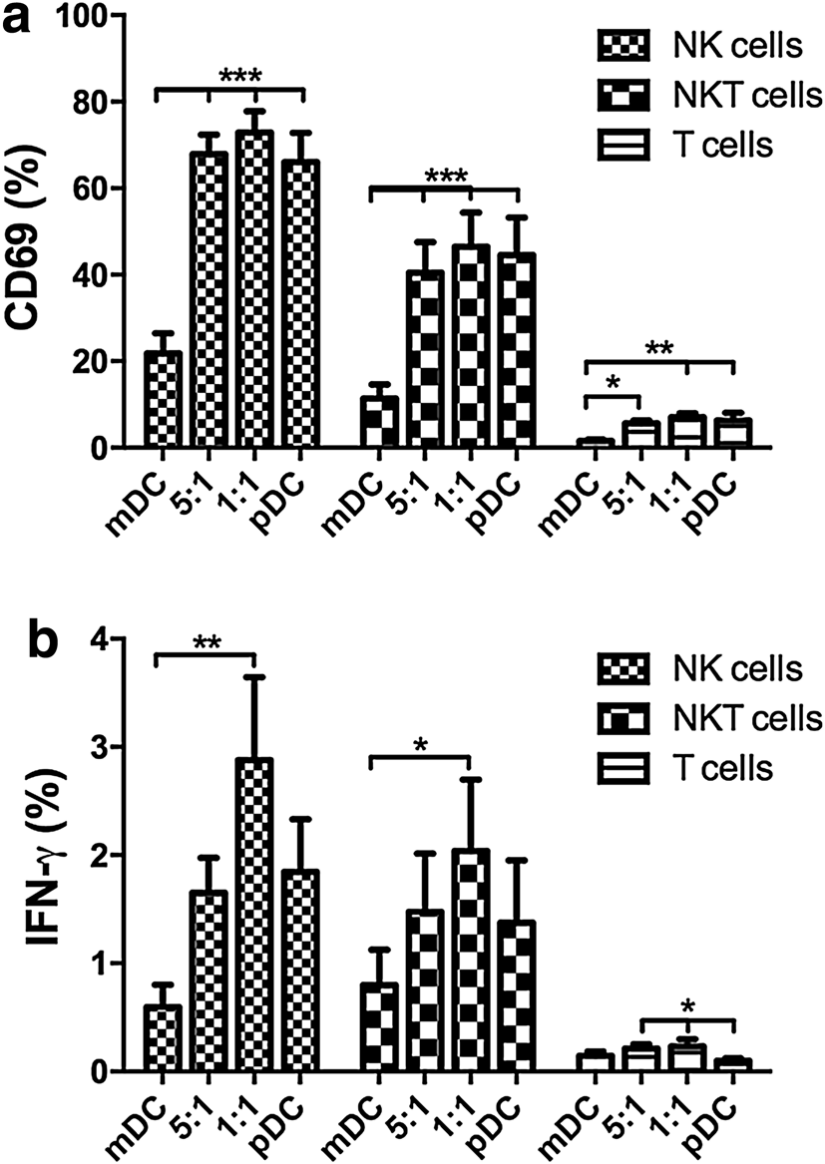
pR-activated pDCs, but not mDCs, are able to activate innate immune cells. mDCs and pDCs were pR-activated overnight, either alone or in 5:1 or 1:1 ratios and thereafter co-cultured another 24 h with allogeneic PBLs, whereafter the upregulation of activation marker CD69 and cell type-specific IFN-γ production was determined by flow cytometry. Mean expression ± SEM of **a** CD69 and **b** IFN-γ from six PBL donors was determined. Statistical differences compared within cell type were analyzed by paired one-way ANOVA with Bonferroni’s multiple comparison test and significance is indicated by *(*p* < 0.05), **(*p* < 0.01), ***(*p* < 0.001). All unlabeled comparisons were statistically non-significant

To investigate if innate immune cells also can be activated by pR-stimulated DCs, their activation status was examined upon co-culture with mDC and/or pDCs (Fig. 4). Autologous mDCs and pDCs were cultured in the same ratios as in Fig. 2 and allogeneic PBLs were added after overnight stimulation for another 24 h. The cell type-specific expression of CD69 and IFN-γ was thereafter investigated. Both NK cells and NKT cells upregulated CD69 after overnight co-culture with pDCs or pDCs co-cultured with mDCs (Fig. 4a). However, in the presence of mDCs alone, only a minor fraction of cells expressed this activation marker. Stimulated pDCs were also able to induce IFN-γ production in innate immune cells, with the highest levels being expressed in cells from the 1:1 ratio mDC–pDC co-culture, while only background levels were detected in cells stimulated with only with mDCs (Fig. 4b).

### Soluble factors produced by pR-stimulated pDCs reduces the proliferative response of T cells

**Fig. 5:**
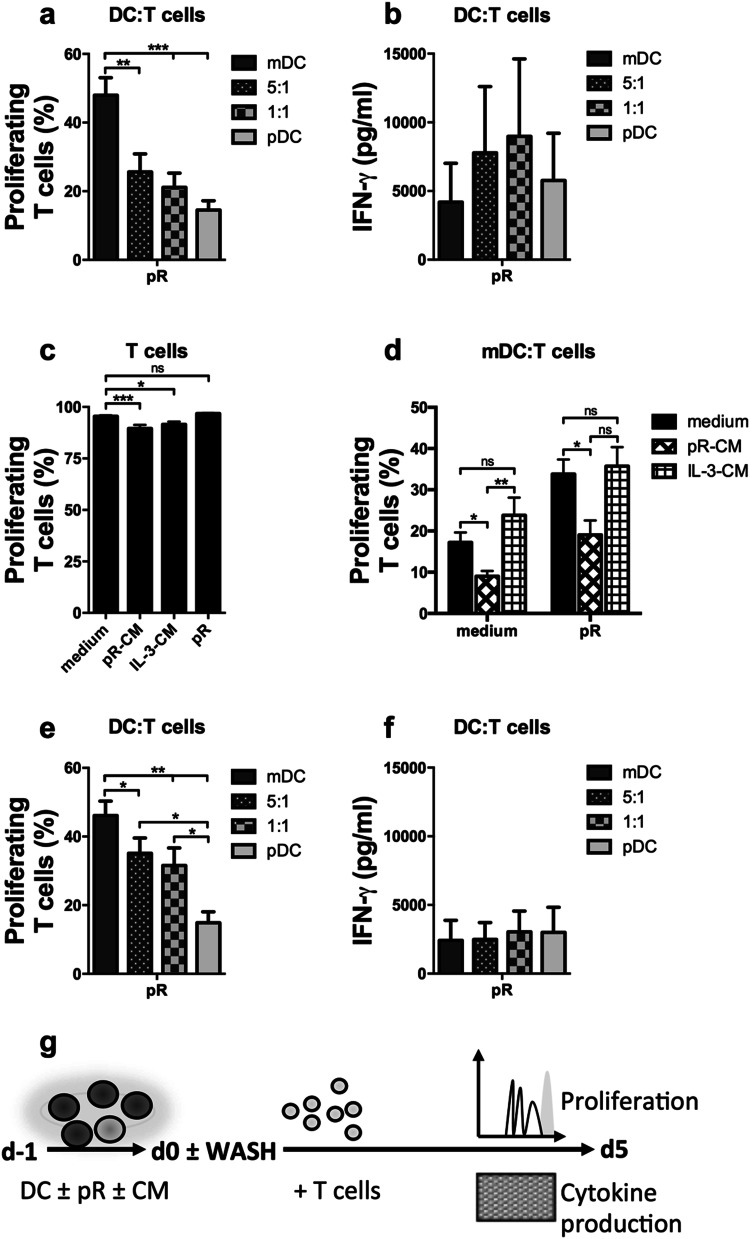
Secreted factors from pR-activated pDCs reduce the proliferation of stimulated T cells but increase their ability to produce IFN-γ. T cells were stimulated with overnight-activated mDCs and pDCs, cultured either alone or in 5:1 or 1:1 ratios, or with anti-CD3/anti-CD28 beads in the presence of indicated stimuli. The proliferative responses were investigated after 5 days by flow cytometry. Secretion of IFN-γ at day 4 was measured by ELISA. **a** Mean percentage ± SEM proliferating T cells induced by six DC donors. **b** Mean levels of secreted IFN-γ ± SEM from seven DC donors at day 4 of culture. **c** Mean percentage ± SEM proliferating anti-CD3/anti-CD28-activated T cells treated with medium alone, pR-CM, IL-3-CM, or freshly made pR complexes from six donors. **d** Mean percentage ± SEM proliferating T cells induced by three to seven mDC donors in the presence of medium alone, pR-CM, or IL-3-CM. **e** Mean percentage ± SEM proliferating T cells induced by six DC donors that were washed three times before addition of T cells. **f** Mean levels of secreted IFN-γ ± SEM at day 4 from seven washed DC donors. **g** Illustrative drawing of experimental set-up. Statistical differences were analyzed by paired one-way ANOVA or, for (**d**), ordinary ANOVA with Bonferroni’s multiple comparison test and significance is indicated by *(*p* < 0.05), **(*p* < 0.01), ***(*p* < 0.001), or ns (non-significant). All unlabeled comparisons in figures **a, b, e**, and **f** were statistically non-significant

To explore the effect of IFN-α and IL-12 on T-cell activation in more detail and considering the prominent effect of pDCs on innate immune cells, subsequent studies of adaptive responses were performed with isolated T cells. Allogeneic T cells were added to pR-activated DCs. mDCs induced a robust proliferative T-cell response. Having pDCs in the culture reduced this response (Fig. 5a, g), a pattern that was not observed for the IFN-γ release (Fig. 5b, g).

Since rIFN-α in Fig. 3c was shown to inhibit mDC-induced T-cell proliferation, the impact of released factors from pR-treated pDCs was investigated on stimulated T cells. Conditioned medium (CM) was made by stimulating pDCs overnight with either pR complexes (pR-CM) or IL-3 (IL-3-CM), and the supernatant from several donors was collected and pooled. In the presence of pR-CM, anti-CD3/anti-CD28-stimulated T cells displayed a reduced proliferative response compared to untreated activated cells (Fig. 5c). This was less prominent for cells cultured with IL-3-CM or pR complexes. In a similar manner, the mDC-induced T-cell proliferation was markedly less in the presence of pR–CM than of IL-3-CM or untreated pR-stimulated mDCs (Fig. 5d), despite that pR–CM-treated pR-stimulated mDCs expressed higher levels of maturation markers than pR treatment alone (Suppl. Figure 4a). The involvement of IFN-α in the reduced proliferative response was confirmed by adding the cytokine to pR-stimulated mDCs together with the allogeneic T cells (Suppl. Figure 4b). To circumvent the observed inhibitory effect of IFN-α on T-cell proliferation, the DC cultures were thoroughly washed just before the addition of allogeneic T cells. This increased the proliferative response of T cells activated by mDCs co-cultured with pDCs, but reduced IFN-γ secretion (Fig. 5e, f).

## Discussion

In this study, we have investigated the effect of type I IFNs on human blood-derived mDC maturation and function. This is an important question in light of clinical treatment modalities involving type I IFNs and agents stimulating high levels of it. However, the answers found in literature are partly contradictory, where the addition of type I IFNs to myeloid DCs has been shown to both inhibit [18–20] and promote [17, 21, 22] IL-12p70 production, and mostly does not investigate the effect of naturally produced type I IFNs but rely on selected recombinant proteins.

We here demonstrate that IFN-α has a beneficial impact on mDC maturation and IL-12p70 production, but impair T-cell proliferation, while recombinant IL-12p70 had no effect on pDC maturation or cytokine production. Type I IFN signaling is required for pR-mediated mDC maturation, since blocking experiments abolished the upregulation of maturation markers. Adding rIFN-α to mDCs significantly enhanced pR-induced IL-12p70 release, corresponding to previous observations in murine bone marrow-derived DCs [17] and human moDCs [17, 21, 22]. However, type I IFNs alone do not provoke IL-12 production, but need to be combined with an IL-12p70-inducing stimulus [21, 34]. This, on the other hand, does not hold true for DC maturation. When treating unstimulated mDCs with increasing doses of rIFN-α, we observed that the highest dose used results in an even higher expression of maturation markers than for pR-stimulated DCs. The ability of type I IFNs to mature DCs is supported by literature [23, 34–36].

The presence of pDCs strongly enhanced the maturation response of mDCs in pR-activated co-cultures. In addition, despite reduced total numbers of mDCs, the IL-12p70 secretion remained high in the co-cultures, indicating an increased capacity of the co-cultured mDCs to produce IL-12p70. For pDCs, the production of IFN-α correlated much stricter with the total number of cells in the culture. Several studies on mDC–pDC crosstalk have highlighted the importance of pDCs to fully potentiate the mDCs [14]. Both cell–cell interaction and secreted factors have been described as mediators of this crosstalk, but type I IFNs emerge as a key factor [15, 37, 38]. Confirming the stimulatory ability of pDC-secreted factors, we could induce a strong maturation response in mDCs treated with conditioned medium from pR-activated pDCs. However, since pR complexes mature both pDCs and mDCs, we cannot fully exclude that this effect is not partly due to remaining complexes in the supernatant, although pR complexes incubated overnight are strongly reduced in their stimulatory ability (data not shown) and their half-time is reportedly a few hours [27].

Type I IFNs have been shown to polarize a Th1 response in naïve T cells [39], although it is questioned whether they fully can substitute the effect of IL-12p70 or if additional cytokine signaling is needed [40, 41]. An elegant study by Ramos et al. suggested that IFN-α and IL-12p70 induce varied effector functions in stimulated CD8^+^ T cells, where IL-12p70 signaling promoted fast dividing T cells with an effector-memory phenotype. IFN-α, on the other hand, reduced proliferation in responding cells and rather stimulated a central-memory phenotype [42]. Importantly, the strength of the T-cell receptor signaling determine which phenotype predominantly develops [42–44], possibly explaining the distinct reduction in T-cell proliferation we observe in MLRs with IFN-α- or pR-CM-treated mDCs, but much less when T cells are stimulated with anti-CD3/anti-CD28 beads. One could speculate that reducing the dose of the highly stimulatory activation beads would make the T cells more sensitive to the polarizing effect of type I IFNs and hence render a similar dose–response pattern as in the MLRs. Further support of the IFN-α-mediated polarization is found in a recent study by Willemen *et al*., where moDCs engineered to secrete high levels of IFN-α had a reducing effect on both CD4^+^ and CD8^+^ T-cell proliferations in an IFN-α-dependent manner. Interestingly, antigen-specific T-cell activation and IFN-γ production was markedly increased in the presence of IFN-α, despite their reduced proliferative response [33]. This correlates well with our observations of a reduced proliferative T-cell response but increased IFN-γ secretion in MLRs with co-cultured DCs. In addition, thorough washing of co-cultured DCs before addition of allogeneic T cells increased the T-cell proliferation, but reduced IFN-γ production, further supporting the direct effect of type I IFNs on T cells.

Although washing the DC cultures increases T-cell proliferation, removing the secreted factors does not fully restore the proliferative response induced by mDCs alone. This might be explained by a reduced total number of mDCs, since pR-stimulated pDCs are modest inducers of T-cell proliferation, but it might also be a symptom of the enhanced maturation response detected for mDCs in the co-cultures. Washing also removes the elevated levels of pro-inflammatory cytokines and IL-12p70 from the culture and due to the late kinetics of 24-h activation, these factors are not replaced. Several studies supporting the IFN-α-mediated enhancement of IL-12-production also indicate a time dependence for this effect, where addition of type I IFNs at a later point in mDC maturation inhibits further production of IL-12p70 [20, 21]. Hence, an earlier wash of the DC cultures and addition of isolated T cells would likely be more beneficial.

In this study, we have investigated two different ratios of mDC–pDC co-culture. However, surprisingly few differences between the groups are detected. Even a low number of pDCs can significantly enhance mDC maturation and affect T-cell proliferation and activation. Indeed, the only set-up where an equal ratio between mDC and pDCs was beneficial was when studying the innate immune cell activation. IFN-α alone had the ability to activate NK cells in a dose-dependent manner, and stimulated pDCs induced an even greater effect. Stimulated mDCs alone, however, were poor inducers of innate immune cell activation, even though IL-12p70 is a known activator of both NK and NKT cells [45, 46], and the presence of pDCs was required to upregulate the activation marker CD69. However, pDCs alone were not sufficient to significantly increase IFN-γ production in innate immune cells. Instead, the 1:1 combination with mDCs was necessary to induce peak activation of both NK and NKT cells. In this culture, the IFN-α levels are still high, while IL-12p70 is also present, which implies that synergistic effects on innate immune activation can be achieved by combining the two DC subsets and thereby supplying both IFN-α and IL-12p70. Nevertheless, innate immune cells have been shown to respond to several other factors provided by activated DCs [10], and we have not excluded the involvement of additional factors in this crosstalk.

To conclude, we here confirm previous studies describing beneficial effects of type I IFNs on mDC maturation and IL-12p70 production [17, 21, 22]. We additionally show that activated type I IFN-producing pDCs also have this effect on mDCs and are highly beneficial for the activation of innate immune cells. The observed reduced proliferative response of T cells stimulated by co-cultured DCs is mostly dependent on the inhibitory effect of secreted factors, rather than a reduced stimulatory effect of co-cultured mDCs. This might explain why some studies have ascribed type I IFNs a negative effect on the functionality of mDCs [18, 20, 21]. It also indicates that *ex vivo*-stimulated DCs will still be functional *in vivo* if the cells are stimulated for a shorter time and washed before administration to the patient. The ratio between the co-cultured DC subsets is less important for the functional effects, and even a minor fraction of activated pDCs has a positive effect on both innate immune cell and T-cell activations. Indeed, melanoma patients treated with a pDC-based cellular vaccine displayed an IFN signature, which even was detected in patients receiving the lowest dose cells, indicating that stimulated human pDCs are highly potent also in vivo [4]. Since pDCs are less prevalent than mDCs in human blood, we conclude that cellular immunotherapies based on both primary DC subsets can utilize all cells isolated, despite a lower ratio of pDCs, and still be predicted highly functional.

### Electronic supplementary material

Below is the link to the electronic supplementary material.

Supplementary material 1 (PDF 321 KB)

## Abbreviations

CM: Conditioned medium
mDC: Myeloid dendritic cell
moDC: Monocyte-derived dendritic cell
pDC: Plasmacytoid dendritic cell
pR: Protamine-RNA
rIL-12: Recombinant interleukin-12p70
