# Supplementary material for: Naturally produced type I IFNs enhance human myeloid dendritic cell maturation and IL-12p70 production and mediate elevated effector functions in innate and adaptive immune cells
Source: Cancer Immunol Immunother. 2018 Jul 13;67(9):1425–36. doi: 10.1007/s00262-018-2204-2 (PMC6132867; doi:10.1007/s00262-018-2204-2)
Supplement: Supplementary file 1 — Supplementary material 1 (PDF 321 KB) [file 262_2018_2204_MOESM1_ESM.pdf]

Supplementary Figure 1

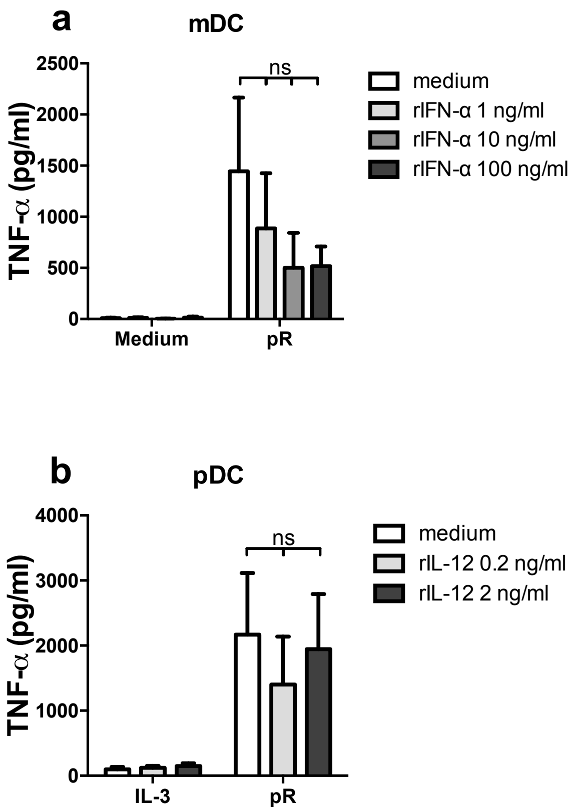

Purified mDCs and pDCs were stimulated overnight with medium alone/IL-3 or pR complexes in the presence or absence of increasing concentration recombinant IFN-α or IL-12p70, respectively. TNF-α release into supernatant was analyzed with ELISA. (**a**) Average secreted levels of TNF-α ± SEM from 9 mDC donors in the presence or absence of increasing levels (1; 10; and 100 ng/ml) of rIFN-α. and (**b**) Average secreted levels of TNF-α ± SEM from 6 pDC donors in the presence or absence of increasing levels (200 and 2000 pg/ml) of rIL-12p70. Statistical differences compared to untreated medium/IL-3 or pR-activated controls were analyzed by paired one-way ANOVA with Bonferroni's multiple comparison test.

Supplementary Figure 2

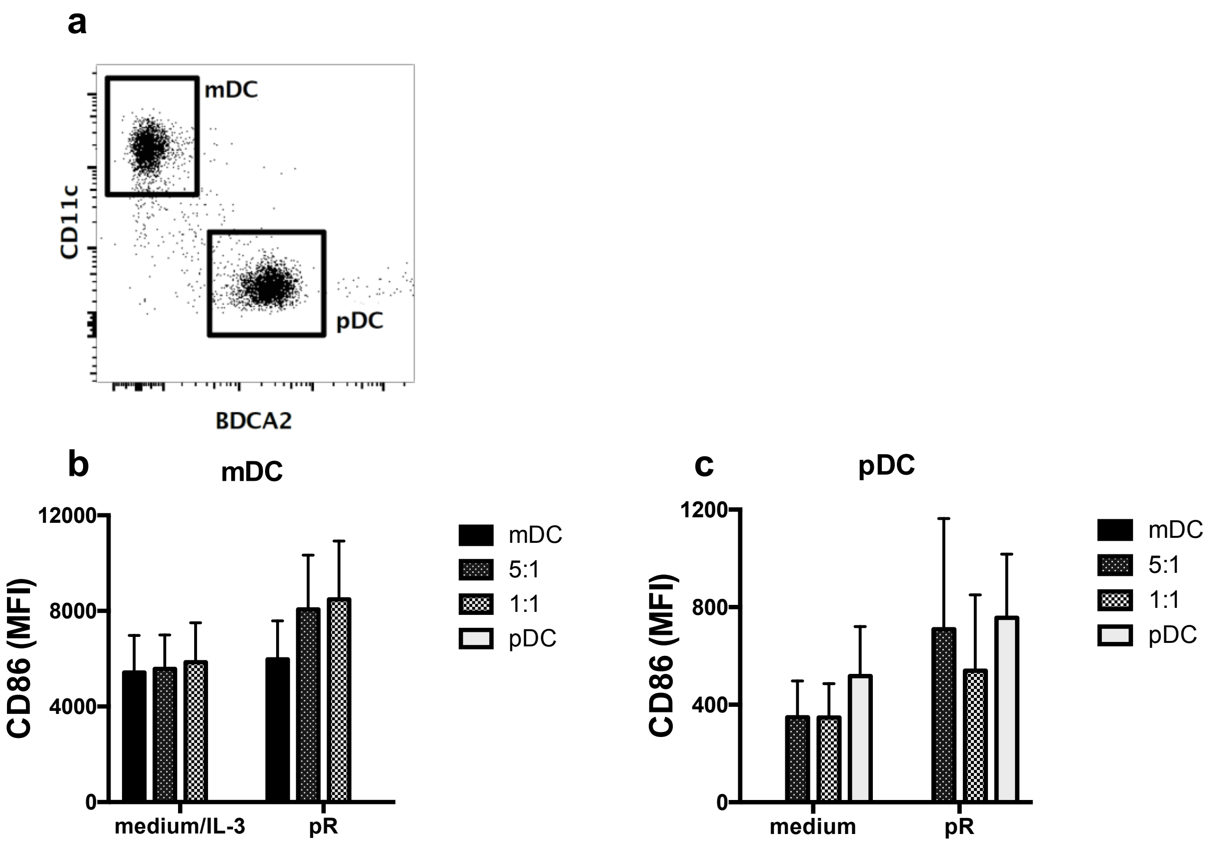

Purified autologous mDCs and pDCs were cultured overnight either alone or together in a 5:1 or 1:1 ratio in the presence of medium alone/IL-3 or pR complexes. The total number of cells was kept constant between the cultures. Upregulation of maturation marker CD86 was evaluated by flow cytometry. (**a**) Representative figure of gating strategy for identification of $CD11c^{+}$ mDCs and $BDCA2^{+}$ pDCs. Average MFI expression levels of CD86 ± SEM on (**b**) mDCs or (**c**) pDCs from 5 donors cultured either alone or combined at a 5:1 or 1:1 ratio in the presence or absence of stimuli.

Supplementary Figure 3

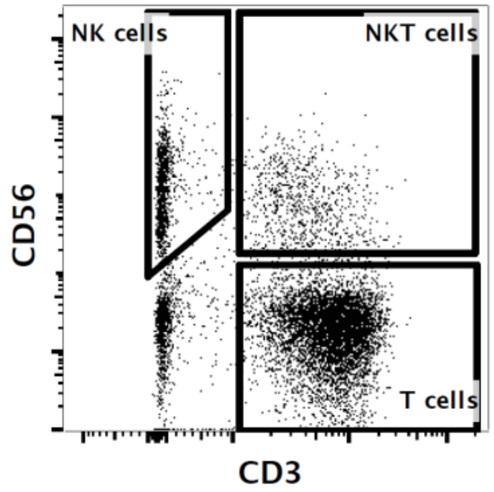

Representative figure of gating strategy for identification of $CD56^{+}CD3^{-}$ NK cells, $CD56^{+}CD3^{+}$ NKT cells, and $CD56^{-}CD3^{+}$ T cells in co-cultures of activated DCs and allogenic PBLs

Supplementary Figure 4

a

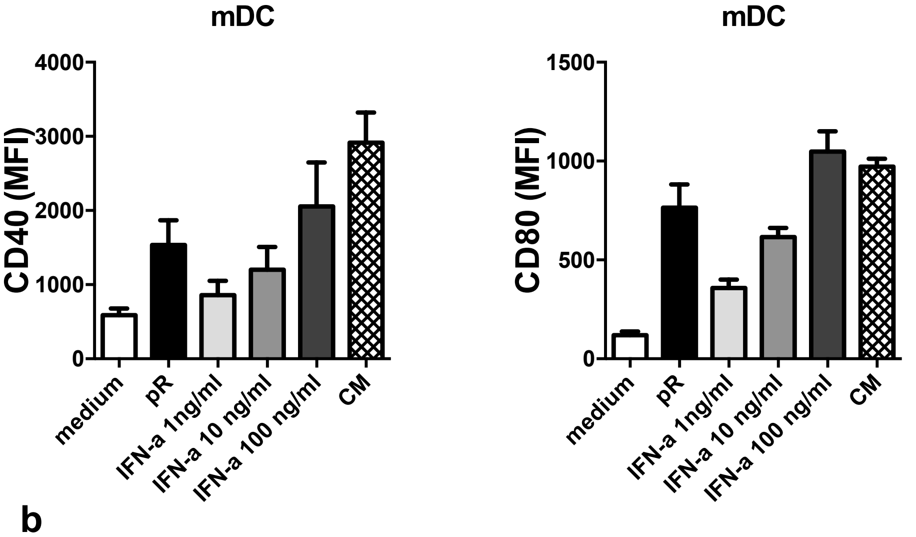

b

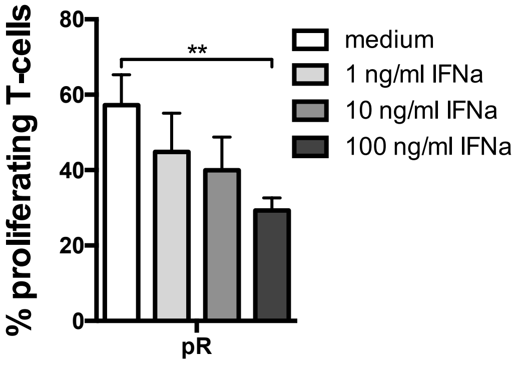

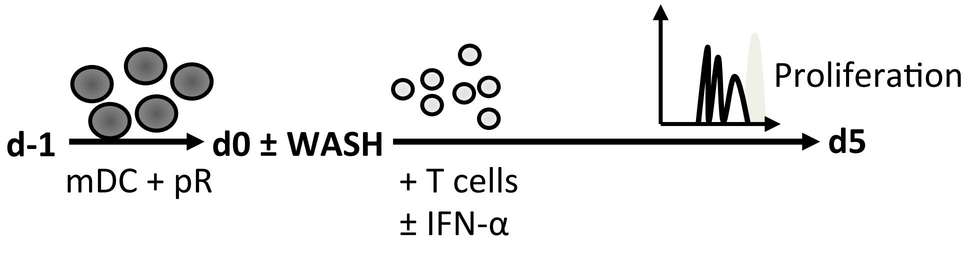

(**a**) Purified mDCs were activated overnight with medium alone, pR complexes, increasing levels (1; 10; and 100 ng/ml) rIFN-α, or pR-CM. The mean expression ± SEM of CD40 and CD80 was investigated with flow cytometry. (**b**) Purified mDCs were activated overnight with pR complexes. Mean percentage ± SEM proliferating T cells induced by 6 DC donors that were washed 3 timed before addition of T cells and increasing levels (1; 10; and 100 ng/ml) of rIFN-α. Statistical differences were analyzed by paired one-way ANOVA with Bonferroni's multiple comparison test and significance is indicated by ** ($p<0.05$). (**c**) Illustrative picture of (**b**).
